# Sphingolipid serum profiling in vitamin D deficient and dyslipidemic obese dimorphic adults

**DOI:** 10.1038/s41598-019-53122-4

**Published:** 2019-11-13

**Authors:** Nasser M. Al-Daghri, Enrica Torretta, Pietro Barbacini, Hannah Asare, Cristian Ricci, Daniele Capitanio, Franca Rosa Guerini, Shaun B. Sabico, Majed S. Alokail, Mario Clerici, Cecilia Gelfi

**Affiliations:** 1 Prince Mutaib Chair for Biomarkers of Osteoporosis, Biochemistry Department, College of Science, King Saud University, Riyadh, 11451 Saudi Arabia; 2 Department of Biomedical Sciences for Health, University of Milan, Segrate-Milano, Italy; 3 Centre of Excellence for Nutrition (CEN), Potchefstroom, 2531 South Africa; 4 IRCCS Istituto Ortopedico Galeazzi, Milano, Italy; 5 IRCCS Fondazione Don Carlo Gnocchi, Milano, Italy; 6 Department of Physiopathology and Transplants, University of Milano, Milano, Italy

**Keywords:** Metabolic disorders, Molecular medicine

## Abstract

Recent studies on Saudi Arabians indicate a prevalence of dyslipidemia and vitamin D deficiency (25(OH)D) in both normal weight and obese subjects. In the present study the sphingolipid pattern was investigated in 23 normolipidemic normal weight (NW), 46 vitamin D deficient dyslipidemic normal weight (-vitDNW) and 60 vitamin D deficient dyslipidemic obese (-vitDO) men and women by HPTLC-primuline profiling and LC-MS analyses. Results indicate higher levels of total ceramide (Cer) and dihydroceramide (dhCers C18–22) and lower levels of total sphingomyelins (SMs) and dihydrosphingomyelin (dhSM) not only in -vitDO subjects compared to NW, but also in –vitDNW individuals. A dependency on body mass index (BMI) was observed analyzing specific Cer acyl chains levels. Lower levels of C20 and 24 were observed in men and C24.2 in women, respectively. Furthermore, LC-MS analyses display dimorphic changes in NW, -vitDNW and –vitDO subjects. In conclusion, LC-MS data identify the independency of the axis high Cers, dhCers and SMs from obesity *per se*. Furthermore, it indicates that long chains Cers levels are specific target of weight gain and that circulating Cer and SM levels are linked to sexual dimorphism status and can contribute to predict obese related co-morbidities in men and women.

## Introduction

Obesity is a globally expanding pathology and up to 58% of the world’s adult population is expected to be overweight or obese by 2030^1^. This condition is associated with vitamin D deficiency and is more common in women (11% of men and 15% of women were obese in 2014)^2–4^. Although a causal genetic association between obesity and vitamin D (25(OH) D) deficiency has been described^5^, the reasons for this association have not yet been clarified and could include a number of factors, such as differences in dietary intake or sun exposure, a decreased vitamin D bioavailability or altered vitamin D metabolism^6^. Furthermore, low plasma levels of vitamin D have been associated with obesity-related health complications, such as insulin resistance, type 2 diabetes and dyslipidemia^7–9^.

A recent differential proteomic study of sera of obese men and women from Saudi Arabia, in whom low vitamin D concentrations were present, identified a number of proteins that are differentially expressed in obese compared to lean weight people; these proteins belong to different pathways, including lipid metabolism, vitamin D function and immunity/inflammation^10^. Serum profiling by MALDI mass spectrometry on these same individuals indicated that increased inflammation and altered lipid metabolism were present in obese subjects^11^. Furthermore, polymorphisms of the synaptosomal-associated protein 25 (SNAP-25) gene associated with insulin resistance have been identified in obese individuals; in particular, the SNAP25 rs363050 (G) allele was shown to result in a reduced expression of SNAP25, associated with altered glycemic parameters^12^.

Recently, increased attention has been focused on circulating levels of ceramide (Cer) and ceramide derivatives associated with obesity and liver steatosis, highlighting the relationship between obesity and circulating lipids^13–16^.

Thus, obese insulin-resistant individuals are characterized by significantly increased plasma Cer concentration, compared to lean, insulin sensitive subjects^14^. The dependence of Cer synthesis on saturated fats can provide a direct link between sphingolipids, dyslipidemia and insulin signaling^17,18^. Furthermore, it has been described that sphingolipid (SL) concentration, in association with adiponectin, IL-6 and insulin resistance, contributes to sexual dimorphism of the adipose tissue distribution in humans^19^.

Recent data indicate that plasma Cer is mainly concentrated in VLDL and LDL particles, that LDL-bound Cer promote inflammation and insulin resistance in skeletal muscle^20^, and that Cer levels correlate with LDL levels and cardiovascular risk^21^. To make the picture more complex, quali /quantitative results of SLs are characterized by high variability due to their peculiar chemico-physical properties and their wide serum dynamic range. In serum, sphingomyelins (SM) account for 87% of all SLs, and Cer account for 2.8%^22^. Recently, thanks to the development of a sphingosine-1-phosphate (S1P)-specific monoclonal antibody^23^, quantitative measurements of S1P plasma levels could be obtained in high fat diet, ob/ob mice and obese patients; results indicated a direct association between S1P levels and obesity^13^.

Nevertheless, an assessment of levels of SLs related to vitamin D deficiency and dyslipidemia and their relationship with obesity is lacking; these data are needed to shed light on the correlations between these factors. Previously, Cer levels were determined considering obese dyslipidemic compared to normal weight normolipidemic without considering dyslipidemic normal weight subjects^14,24^.

This study, based on the combination of a single phase extraction method with a HPTLC-primuline-profiling and LC-MS analyses was designed with the goal of analyzing SLs changes in dyslipidemia and vitamin D deficiency, to get better insight into the contribution of SLs in obesity. To this end, we investigated SLs levels in three groups of Saudi Arabians: normolipidemic normal weight (NW), vitamin D deficient dyslipidemic normal weight (-vitDNW) and vitamin D deficient dyslipidemic obese (-vitDO) subjects. Each group was composed by men and women, making possible to assess gender differences in the three groups. Comparison between vitamin D deficient lean and obese subjects allowed for analyzing SLs patterns in obesity independently from dyslipidemia, thus verifying whether these patterns are linked to weight gain and/or to chronic inflammation.

## Results

### Subjects’ general characteristics and clinical parameters assessment

Serum samples were collected from 23 normolipidemic normal weight (NW) controls (M/F-15/8), 46 vitamin D deficient dyslipidemic (HDL < 1 mmol/L; TG > 2.3 mmol/L) normal weight (-vitDNW) subjects (M/F-23/23) and from 60 vitamin D deficient dyslipidemic obese (-vitDO) subjects (M/F-25/35). Epidemiologic data and median serum levels of total cholesterol (CHL), glucose (GLU), high density lipoprotein-CHOL (HDL), triglycerides (TRG) and 25(OH)D of enrolled Saudi individuals are summarized in Table 1. Gender and age composition were homogeneous across groups composed of normal weight and obese subjects (U-test p-value = 0.64, *χ*^2^ p-value = 0.15 for age and gender respectively). -vitDNW subjects were characterized by dyslipidemia, with lower levels of HDL and higher levels of TRG and total cholesterol compared to NW (U-test p-value < 0.001 for HDL, TRG and total cholesterol), and with higher levels of TRG and lower levels of HDL compared to -vitDO (U-test p-value = 0.019 and 0.006 for HDL and TRG respectively).

**Table 1:** Characteristics of participants. Continuous variables were described by median and interquartile range, categorical variables were reported as counts and percentages.

	Men			Women		
	Normolipidemic normal weight (NW)	Dyslipidemic normal weight (-vitDNW)	Obese (-vitDO)	Normolipidemic normal weight (NW)	Dyslipidemic normal weight (-vitDNW)	Obese (-vitDO)
N (%)	15 (11.6%)	23 (17.8%)	25 (19.4%)	8 (6.2%)	23 (17.8%)	35 (27.1%)
Age (year)	39 (35, 44)	47.0 (32.0, 59.0)	44.0 (31.0, 56.0)	39 (26, 48.5)	45.0 (35.0, 55.0)	45.0 (40.0, 56.0)
BMI (kg/m^2^)	23.3 (22.0, 23.9)	23.4 (22.2, 24.6)	36.7 (34.7, 39.1)	20.7 (19.4, 22.9)	25.6 (24.0, 26.0)	39.9 (37.6, 42.6)
WC (cm)	—	88.9 (81.0, 93.0)	117 (113, 123)	—	91 (82, 102)	105 (98.2, 112)
BP (cm)	—	86.0 (39.0, 98.0)	116 (53, 126)	—	103 (94, 106)	121 (115, 129)
SBP (mmHg)	—	122 (113, 130)	130 (120, 141)	—	125 (110, 130)	130 (117, 132)
DBP (mmHg)	—	71.5 (68.0, 76.0)	80.0 (68.5, 90.0)	—	80.0 (70.0, 82.0)	80.0 (72.0, 83.0)
GLU (mmol/l)	—	6.3 (5.0, 9.9)	7.0 (5.9, 14.4)	—	6.2 (4.7, 11.8)	8.0 (6.0, 11.6)
CHL (mmol/l)	4.6 (4.3, 4.8)	4.9 (4.4, 6.1)	5.4 (4.7, 6.0)	4.4 (4.0, 4.6)	5.4 (4.7, 6.2)	5.4 (4.7, 6.0)
HDL (mmol/l)	1.76 (1.71, 1.86)	0.7 (0.6, 0.8)	0.8 (0.6, 0.8)	1.47 (1.45, 1.63)	0.7 (0.6, 0.8)	0.8 (0.7, 0.9)
TRG (mmol/l)	0.90 (0.7, 1.50)	3.4 (3.0, 4.2)	3.2 (2.9, 3.9)	0.78 (0.65, 0.91)	3.4 (2.8, 4.1)	3.0 (2.7, 3.3)
25(OH) D (nmol/l)	75.4 (65.7, 88.9)	32.7 (21.9, 40.8)	34.0 (26.6, 37.6)	78.9(65.2, 84.2	22.0 (14.4, 37.0)	31.0 (21.5, 42.0)

*Notes:* **BMI** Body Mass Index, **WC** Waist Circumference, **BP** Buttocks Perimeter, **SBP** Systolic Blood Pressure, **DBP** Diastolic Blood Pressure, **GLU** Glucose, **CHL** Cholesterol, **HDL** High Density lipo-protein, **TRG** triglycerides.

### Variations in ceramide and sphingomyelin profiles

To identify variations in total ceramide (Cer) and total sphingomyelin (SM) levels associated to dyslipidemia, vitamin D deficiency and obesity, HPTLC-primuline profiling was performed on SLs extracted from NW (n = 15), -vitDNW (n = 23) and -vitDO (n = 25) men and from NW (n = 8), -vitDNW (n = 23) and -vitDO (n = 35) women. After separation on HPTLC plates, SL bands were compared by a profiling approach based on HPTLC-densitometry and FDIC (fluorescence detection by intensity changes) emission after primuline staining (data shown in Fig. S1).

From primuline stained HPTLC plates, bands corresponding to Cer and SM were identified by Rf comparison with standards, carrying a variation coefficient (CV) of 6–7%; bands with Rf = 0.923, Rf = 0.858, Rf = 0.176 and Rf = 0.152 respectively, were attributed to Cers (C20-C24), Cers (C14-C18), SMs (C20-C24) and SMs (C14-C18) (Fig. S2).

**Figure 1:**
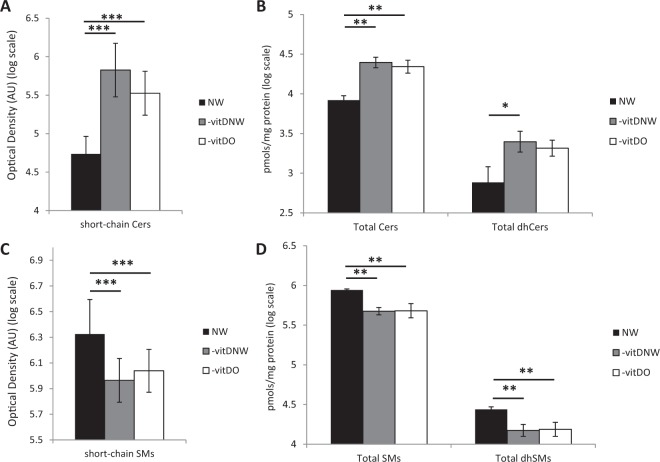
(**A**) Ceramides (Cers) (C14-C18) in sera from normolipidemic normal weight (NW) (n = 15), vitamin D deficient dyslipidemic normal weight (-vitDNW) (n = 46) and vitamin D deficient dyslipidemic obese (-vitDO) (n = 60) subjects by primuline /HPTLC densitometry. (**B**) Total Cer and dhCer in sub-pooled sera from NW vs –vitDNW vs –vitDO subjects by LC-MS analysis. (**C**) Sphingomyelins (SMs) (C14-C18) in sera from NW, -vitDNW and –vitDO subjects by primuline /HPTLC densitometry. (**D**) Total SM and dhSM in sub-pooled sera from NW vs –vitDNW vs –vitDO subjects by LC-MS analysis. Statistical analysis was performed by ANOVA test, with Tukey post-hoc test. Data are expressed in log scale and reported as mean ± SD.

Primuline quantitative staining revealed a statistically significant increase in Cers (C14-C18) levels in -vitDNW and -vitDO compared to NW control subjects (omnibus p-value < 0.001, NW vs -vitDO p-value < 0.001, NW vs -vitDNW p-value < 0.001) (Fig. 1A). To get better insight into the acyl chain qualitative and quantitative composition of the sphingolipidome, LC-MS analyses were conducted on 4 sub-pools per group, both for men and women (24 total sub-pools). Total Cer levels were higher in –vitDNW and in –vitDO compared to NW (p-value < 0.01) (Fig. 1B), as the Cers C16:0, C20:0, C22:0, C22:1, C24:0, C24:1, C24:2, whereas Cers C20:0 and C24:1 were lower in –vitDO men compared to –vitDNW men, while only C24:2 was lower in –vitDO women compared to -vitDNW women (Fig. S3,A,B). Notably, total dihydroceramide (dhCer) levels were higher in –vitDNW compared to NW (p-value < 0.05) (Fig. 1B) and dhCers C18:0, C20:0 and C22:0 acyl chain levels were higher both in –vitDNW and in –vitDO compared to NW (Fig. S3,C,D).

Concerning SM levels, NW control subjects showed higher levels of SMs (C14-C18) compared to -vitDNW and -vitDO (p < 0.001) (Fig. 1C). Total SMs, together with total dihydrosphingomyelin (dhSMs) (Fig. 1D), were lower in –vitDNW and in –vitDO compared to NW, with the same trend of HPTLC-primuline profiling. SM specific chains C16:0, C16:1, C18:0, C18:1, C20:1 and C22:1 and dhSM C14:0 and C16:0 were lower compared to NW (Fig. S3 E,F). Data from Cers (C20-C24) and SMs (C20-C24) are reported in Fig. 4S.

### Sex-related variations in sphingolipid profiles

**Figure 2:**
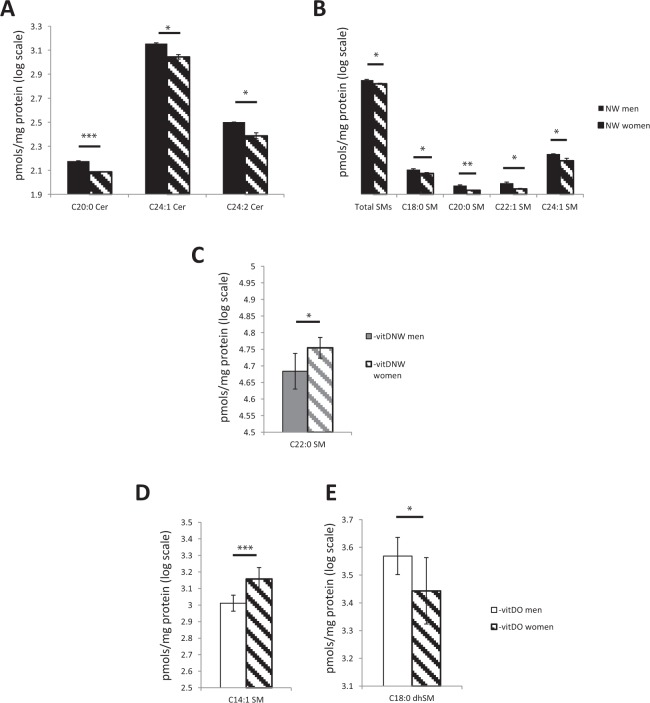
Cer and SM sex-related variations in sub-pooled sera of NW (**A,B**), -vitDNW (**C**), and -vitDO (**D,E**) men and women. Statistical analysis was performed by Student t-test. Data are expressed in log scale and reported as mean ± SD.

**Figure 3:**
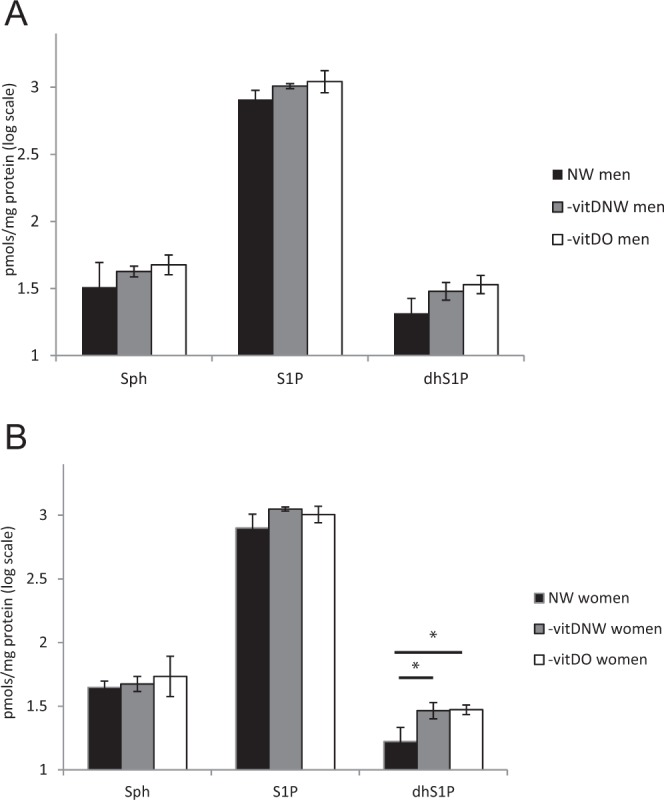
LC-MS profile of sphingosine, sphingosine-1-phosphate (S1P) and dihydrosphingosine-1-phosphate (dhS1P) in sub-pooled sera from NW, –vitDNW and –vitDO men (**A**) and women (**B**). Statistical analysis was performed by ANOVA test, with Tukey post-hoc test. Data are expressed in log scale and reported as mean ± SD.

Gender-specific differences emerged when Cer and SM profiles in men and women were compared (Fig. 3S). To get better insight into sex-related variations, the sphingolipid patterns in men and women were directly compared in NW, -vitDNW and –vitDO. NW men compared to women were characterized by higher levels of Cers (specifically C20:0, C24:1, C24:2 chains) and of SMs (specificallyC18:0, C20:0, C22:1 and C24:1 chains) (Fig. 2A,B). At variance, –vitDNW women compared to men showed higher levels of SM C22:0 (p-value < 0.05) (Fig. 2C). Concerning dhSM and SM in –vitDO men compared to women, dhSM C18:0 was higher in men (p-value < 0.05) (Fig. 2D), whereas SM C14:1 was higher in women (Fig. 2E).

To investigate levels of sphingosine-1-phospahte (S1P), a sphingolipid recently associated with obesity and hypoxia induced obesity^13,25^, sphingosine, S1P, dihydrosphingosine-1-phosphate (dhS1P), and glycosphingolipids as hexosylceramide (HexCer) and dihexosylceramide (diHexCer) (Fig. S5) were analyzed by LC-MS/MS. S1P levels were non statistically significant in –vitDNW and –vitDO compared to NW, both for men and women (Fig. 3A,B). Concerning dhS1P, in women it was significantly higher in –vitDNW and in –vitDO compared to NW (p-value < 0.05) (Fig. 3B).

**Figure 4:**
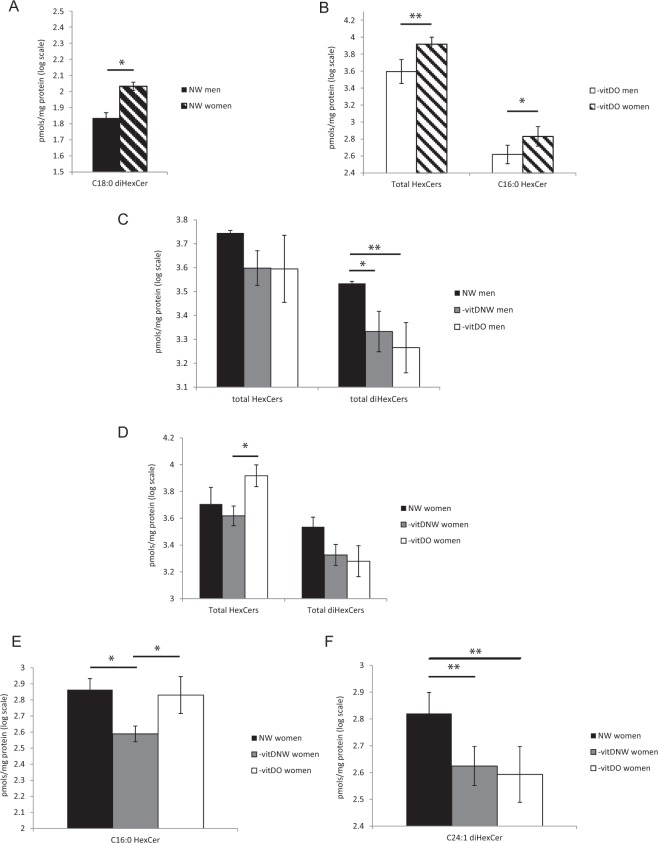
Glycosphingolipid sex-related variations in sub-pooled sera of NW (**A**) and obese (**B**) men and women. LC-MS profiles of total hexosylceramide (HexCer) and dihexosylceramide (diHexCer) in sub-pooled sera of NW, -vitDNW, and -vitDO men (**C**) and women (**D**) are shown, together with LC-MS levels of C16:0 HexCer (**C**) and C24:1 diHexCer (**D**), resulted to be changed in women. Statistical analysis was performed by Student t-test for (**A,B**) and by ANOVA test, with Tukey post-hoc test for (**C–F**). Data are expressed in log scale and reported as mean ± SD.

DiHexCer C18:0 levels were lower in NW men compared to women (p-value < 0.05) (Fig. 4A). In –vitDO subjects, higher levels of total HexCer and HexCer C16:0 were observed in women (Fig. 4B). Data from other sphingolipid classes in men and women are shown in Fig. S6.

In men, results showed that total diHexCer was lower in –vitDNW and –vitDO compared to NW (p-value < 0.05 and <0.01, respectively) (Fig. 4C), whereas in women, total HexCer was higher in –vitDO compared to –vitDNW (p-value <0.01) (Fig. 4D). Concerning levels of specific chains in women: HexCer C16:0 was higher in NW and in –vitDO compared to –vitDNW (p-value < 0.05) (Fig. 4E) whereas DiHexCer C24:1 was lower in –vitDO and in –vitDNW compared to NW (p-value < 0.01) (Fig. 4F).

## Discussion

We investigated SL levels in three groups of Saudi individuals, normolipidemic with normal weight (NW), compared to vitamin D deficient and dyslipidemic subjects who were either obese (-vitDO) or had normal BMI (-vitDNW). Results herein show the presence of a general increase of total Cer in dyslipidemic (both –vitDNW and -vitDO) compared to normolipidemic NW subjects, linking total Cer abundance to dyslipidemia and vitamin D deficiency. Our results confirmed data showing higher levels of total Cer in obese (-vitDO) subjects compared to normolipidemic normal weight (NW) controls^20,26,27^. More interestingly, data herein for the first time highlighted that levels of total Cer are significantly higher not only in obese but also in dyslipidemic normal weight subjects. These results suggest that total Cer increase is associated with 25(OH)D deficiency and hyperlipidemia independently from obesity *per se*.

In countertrend with total Cer, total SM and total dhSM, were lower both in –vitDNW and in –vitDO compared to NW, suggesting also in this case a direct correlation of this class of molecules with vitamin D deficiency and hyperlipemia.

The strength of the present work is the investigation of sera from obese compared to dyslipemic NW and normolipidemic NW subjects that allows a better understanding of the significance of the fine regulation of Cers and SMs. In previous studies, sphingolipids abundance was compared in obese and lean healthy subjects^13,14,28^ or lean normoglycemic^29^ controls, without taking into account dyslipidemia. A recent study indicates the same relationship among 25(OH)D deficiency, total Cer increase and dyslipidemia in Andean children characterized by different BMIs^25^. It could be speculated that in Saudi Arabian subjects, total Cer increment in -vitDNW and –vitDO is related to palmitoyl-CoA /serine and fatty acids de novo biosynthetic route as suggested by the increment of total dhCer and of dhCer specific chains (Fig. S5)^30,31^. Ceramide generation, during HFD-induced obesity, is stimulated by the continuous and excessive supply of FFA from diet and adipose tissue providing substrate for serine palmitoyl transferase and Ceramide synthase CerS(1–6) isoforms^16,32^ increasing plasmatic Cer levels. Notably, total SM and dhSM were decreased in –vitDNW and in –vitDO, suggesting a higher activity of sphingomyelinase in membranes^33^. In animal models, HFD administration stimulates the expression and the activity of both acidic sphingomyelinase (aSMase) and neutral sphingomyelinase (nSMAse)^34,35^. It has been observed that the pharmacological inhibition of aSMase by amitriptyline reduced HFD-stimulated ceramide release in blood^36^.

Little is known regarding SLs behavior during dyslipidemia and vitamin D deficiency. Thanks to their hydrophilicity, sphingolipids circulate in plasma bound to albumin and lipoproteins^37^. Particularly, Cer is present and equally distributed in HDL-C, LDL-C and VLDL-C^22^, while 70% of SM is carried by LDL/VLDL-C and 30% by HDL-C^22^. Interestingly, -vitDNW subjects showed higher levels of total Cer, dhCer and lower levels of total SM and dhSM, compared to normolipidemic NW, suggesting that -vitDNW subjects, independently from obesity, develop a sphingolipid plasma profile that is influenced by LDL-C, HDL-C and TRG levels. It can be speculated that normal-weight subjects, with vitamin D deficiency and dyslipidemia, develop altered plasma SL profiles because of the differential “transport” capacity of lipoprotein, whereas the alterations observed in obesity are related to changes in SLs biosynthesis (e.g., de-novo biosynthesis and sphingomyelinase pathway) (Fig. S5)^21^.

Specific acyl chains of Cer and SM rather than total levels were found at variance in obesity supporting the role of different Cer synthase isoforms in the regulation of SLs production in obesity. Specifically in obese men, we found chains of Cer, namely Cers C20:0 and C24:2 at lower levels and higher levels of dhSM C18:0. Obese women had lower levels of Cer C24:1 and higher levels of SM C16:0. These results pinpoint the relevance of different Cers synthase (1–6) isoforms in the regulation of single acyl chain Cer synthesis in obesity. Our results indicate that differentially abundant acyl-chains Cer are directly associated with increased SM and dhSM specific chains in obese subjects (SM C16:0 in women and dhSM C18:0 in men).

Although sexual dimorphism in body composition is evident in adulthood^38^, sex has been only sporadically considered in metabolomic and lipidomic studies in obesity^39^. Despite increased levels of circulating free fatty acids, women readily oxidize non-esterified plasma fatty acids, increasing ketone bodies production^40^ or lipid re-esterification^41^ and display improved insulin sensitivity.

Results from the present study indicate in men increased levels of Cer C20:0, C24:1 and C24:2 as a characteristic trait of normolipidemic normal-weight subjects confirming results from a larger cohort of Mexican Americans^42^, that attribute to cerS-2 higher levels of long-chain ceramides.

Also SMs characterized dimorphic changes being SM C18:0, C20:0, C22:1 and C24:1 higher in normolipidemic normal-weight men compared to women. These results are in contrast with other studies^43–45^ that found higher levels of SM in women. However, once again, considering dyslipidemic normal-weight and obese women, SM were more abundant^44^, and particularly SM C22:0 and C14:1 were higher compared to men both dyslipidemic normal-weight and obese.

Concerning dhS1P, this molecule is present at higher levels in –vitDNW and –vitDO women compared to NW. It can be hypothesized that this molecule contributes to keep under control levels of dhCer thus subtracting dhCer to total Cer synthesis being levels of total Cer in -vitDNW and -vitDO similarly increased both in men and women.

It can be hypothesized that higher levels of hexosylceramide found in obese women could be related to overexpression of hexosylceramide synthase in adipocytes that suppresses insulin signaling^46^. Hexosylceramide has been described as a possible inducer of plaque inflammation and instability^47^. It must be stated that due to the similarity in the hydrophobic moiety and mass identity, hexosylceramides (glucosylceramides and galactosylceramides) are not easy to separate and quantify by reverse-phase chromatography^48^, therefore observed changes are related to both glucosylceramides and galactosylceramides and further studies will be required to precisely identify the contribution of single species to the total pool and the role of different glycosphingolipids.

Although the use of two approaches to study SLs in serum, the quantitative structure-specific measurements of all molecular species remains challenging.

The novelty of this study is that it demonstrated for the first time that Cer and SM levels correlate directly with dyslipidemia and vitD deficiency independently from BMI and that only specific acyl chains of Cer and SM are directly correlated with obesity *per se*. Furthermore, it highlights the importance of sexual dimorphism in determining circulating SLs levels independently from serum lipids and vitamin D status. Notably, results obtained in normal weight subjects indicated that sex influences serum concentrations of Cer and SM^42^. We confirmed those results and expanded them in the setting of obesity and vitamin D deficiency.

However, the inter-individual variability, the number of samples, the paucity of reference data could be seen as limiting factors in this study. These limitations notwithstanding, it is important to underline that results herein suggest that these putative biomarkers could be used to predict the risk for vitamin D deficient adult subjects to develop associated co-morbidity in obesity.

The possible effect of vitamin D and statins supplementation on Cer levels is currently being investigated in our cohorts to better clarify the role of drugs on SLs concentration.

## Materials and Methods

### Participants and sample collection

Adult male and female Saudis were enrolled from the Vitamin D School Project Database of the Prince Mutaib Chair for Biomarkers of Osteoporosis (PMCO), College of Science, King Saud University (KSU), Riyadh, Kingdom of Saudi Arabia (KSA)^49^. All subjects were grouped according to Body Mass Index (BMI) into normal weight (BMI < 25 kg/m^2^) and obese (BMI ≥ 30 kg/m^2^). The present study conforms to the principles of Helsinki Declaration, and was approved by the Ethics Committee of the College of Science, KSU, Riyadh, KSA (Ref No.15/0502/IRB). All enrolled subjects provided their full informed consent. Serum samples were collected from 23 normolipidemic normal weight (NW) controls, 46 vitamin D deficient dyslipidemic (HDL < 1 mmol/L; TG > 2.3 mmol/L) normal weight (-vitDNW) and from 60 vitamin D deficient obese (-vitDO) subjects, and stored at −80 °C until use. The general characteristics of enrolled subjects are summarized in Table 1. Vitamin D deficiency was defined as circulating serum 25(OH)D < 50 nmol/l (<20 ng/ml)^50^.

### Reagents and Chemicals

Propan-2-one, methanol, 1-butanol, LC-MS grade water, primuline yellow dye, ammonium dihydrogen phosphate, 3,5-Di-tert-4-butylhydroxytoluene (BHT) and ammonium formate were from Sigma-Aldrich (Saint Louis, MO, USA). Ethanol and high performance liquid chromatography (HPLC)-analytical grade chloroform (CHCl_3_) were respectively from J.T. Baker (Center Valley, PA, USA) and Carlo Erba (Cornaredo, MI, Italy). N-lignoceroyl-D-erythro-sphingosine (Cer C24:0) 1,2-Dipalmitoyl-sn-Glycero-3-Phosphocholine (DPPC), Cardiolipin (CL), 1,2-Dipalmitoyl-sn-Glycero-3-Phosphoethanolamine (DPPE), D-glucosyl-ß-1,1′-N-stearoyl-D-erythro-sphingosine-d5 (HexCer), N-lauroyl-D-erythro-sphingosine, N-lauroyl-D-erythro-sphinganine, N-lauroyl-D-erythro-sphingosylphosphorylcholine N-lauroyl-D-erythro-sphinganylphosphorylcholine, D-glucosyl-ß−1,1′-N-lauroyl-D-erythro-sphingosine, D-lactosyl-ß-1,1′ N-lauroyl-D-erythro-sphingosine and C17 d-erythro-dihydrosphingosine-1-phosphate lipids standards were from Avanti Polar Lipids (Alabaster, Alabama, USA). SL standard mixture, containing SM and Sulfatides (SLF) was from Matreya LLC (Pleasant Gap, PA, USA).

### Lipid extraction

Sera samples were extracted according to the procedure of Alshehry *et al*.^51^, with minor modifications, as already described in^52^.

### Sphingolipid analysis

For sphingolipid analysis two different strategies were considered: High performance thin layer chromatography (HPTLC)-primuline profiling were first conducted to analyze all serum samples (from 23 NW, 46 –vitDNW and –vitDO subjects), whereas LC-MS analysis were carried out on 4 sub-pools per group, both for men and women (24 total sub-pools).

The use of primuline as a fluorophore for quantitative purposes was first described by Domínguez *et al*.^53^ as FDIC, i.e. fluorescence detection by intensity changes. FDIC detects fluorescent emission produced by non-specific and electrostatic interactions between the primuline and hydrocarbon chains in the ceramide backbones of sphingolipids^54–57^. In our work, primuline was used for semi-quantitative purposes, to detect changes in ceramides and sphingomyelins levels among NW, –vitDNW and –vitDO men and women.

### HPTLC-Primuline profiling

HPTLC-Primuline profiling analyses were performed according to Torretta E. *et al*.^52^. For each sample, 100 µg of total protein were loaded in duplicate using Linomat 5 semiautomatic TLC spotter (CAMAG, Switzerland).

HPTLC plates were then developed in chloroform/methanol/water 55:20:3 (v/v/v), using Camag Automatic Developing Chamber 2 (CAMAG, Switzerland) Developed plates were sprayed with a solution of primuline yellow dye, 5 mg/100 ml in propan-2-one/water 80:20 (v/v) and dried under a fume hood. Images from stained plates were acquired using Ettan DIGE Imager (GE Healthcare, Chicago, IL, USA). Bands corresponding to Cers (C20-C24), Cers (C14-C18), SMs (C20-C24) and SMs (C14-C18) were compared among NW, -vitDNW and –vitDO, considering primuline as a FDIC (fluorescence detection by intensity changes) fluorophore^53,58^.

### Sphingolipids by LC-MS

For LC-MS analysis, sera were randomly sub-pooled into 4 groups of men and 4 groups of women, homogeneous for biometric parameters and HPTLC quantitative profiles. SL extracts were analyzed in the presence of internal standard^52^, prepared as described by Merrill *et al*.^59^, including an alkaline hydrolysis step to remove phospholipids, and analyzed by Waters Aquity Ultra Performance Liquid Chromatography (UPLC) system connected to a Waters LCT Premier orthogonal accelerated time of flight mass spectrometer (Waters, Millford, MA), operating in positive electrospray ionization mode. Spectra were acquired according to^52^. Positive identification of compounds was based on the accurate mass measurement with an error <5 ppm and its LC retention time, compared to that of a standard (±2%). Mass spectra were analyzed by MassLynx™ 4.1 Software.

LC-MS/MS analyses of sphingosine, S1P and dhS1P were carried out on an Acquity UPLC system coupled with a Xevo TQ-MS triple quadrupole mass spectrometer. The mass spectrometer was operated in the positive ESI mode, and analytes were quantified by multiple reaction monitoring (MRM). Transitions considered were Q1 300.4- > Q3 264.4 for Sph, Q1 380.4 - > Q3 264.4 for S1P and Q1 382.4- > Q3 284.4 for dhS1P.

### Statistical analysis

Participants were grouped according to sex, obesity and dyslipidemia status and their characteristics were described using median and interquartile range, if continuous, and percentages, if categorical (Table 1). Comparison of serum cholesterol and triglycerides were performed using Mann-Whitney U-test. Circulating levels of Cer and SM were transformed as logarithm and were compared by sex, weight group and dyslipidemia status using a generalized linear model (GLM) adjusted for BMI and age. Retro transformed least square means from the GLM models were reported along with their standard errors and comparisons were performed using the Tukey adjustment to control for inequalities of groups sizes^60^. For LC-MS analysis, sera were randomly sub-pooled into 4 matched groups of men and women considering biometric parameter and HPTLC quantitative profiles. The pooling was adopted as a method to reduce the variance among biological groups increasing the power to detect changes when few samples are available and the variance is high^61,62^. All statistical analyses were performed using the SAS software version 9.4. Statistical tests were two tailed and type-I error rate was set at 5% (α = 0.05).

## Supplementary information

Supplementary Information

## Data Availability

The data generated during and/or analysed during the current study are included in this published article (and its Supplementary Information files). When needed, further information are available from the corresponding author on reasonable request.
