# Publisher Correction: Sphingolipid serum profiling in vitamin D deficient and dyslipidemic obese dimorphic adults

**DOI:** 10.1038/s41598-020-58287-x

**Published:** 2020-02-04

**Authors:** Nasser M. Al-Daghri, Enrica Torretta, Pietro Barbacini, Hannah Asare, Cristian Ricci, Daniele Capitanio, Franca Rosa Guerini, Shaun B. Sabico, Majed S. Alokail, Mario Clerici, Cecilia Gelfi

**Affiliations:** 1 Prince Mutaib Chair for Biomarkers of Osteoporosis, Biochemistry Department, College of Science, King Saud University, Riyadh, 11451 Saudi Arabia; 2 Department of Biomedical Sciences for Health, University of Milan, Segrate-Milano, Italy; 3 Centre of Excellence for Nutrition (CEN), Potchefstroom, 2531 South Africa; 4 IRCCS Istituto Ortopedico Galeazzi, Milano, Italy; 5 IRCCS Fondazione Don Carlo Gnocchi, Milano, Italy; 6 Department of Physiopathology and Transplants, University of Milano, Milano, Italy

Correction to: *Scientific Reports* 10.1038/s41598-019-53122-4, published online 13 November 2019

In the original version of this Article, the author Franca Rosa Guerini was incorrectly indexed. This error has now been corrected.

