# Supplementary material for: Sphingolipid serum profiling in vitamin D deficient and dyslipidemic obese dimorphic adults
Source: Sci Rep. 2019 Nov 13;9:16664. doi: 10.1038/s41598-019-53122-4 (PMC6853956; doi:10.1038/s41598-019-53122-4)
Supplement: Supplementary file 1 — Supplementary Information [file 41598_2019_53122_MOESM1_ESM.docx]

Supplementary Information

**Sphingolipid serum profiling in vitamin D deficient and dyslipidemic obese dimorphic adults** Nasser M. Al-Daghri^1^, Enrica Torretta^2§^, Pietro Barbacini^2§^, Hannah Asare^3^, Cristian Ricci^3^, Daniele Capitanio^2,^, Franca Rosa Guerini,^4^ Shaun B. Sabico^1^, Majed S. Alokail^1^, Mario Clerici^4,5^, Cecilia Gelfi^2,6*^

^1^ Prince Mutaib Chair for Biomarkers of Osteoporosis, Biochemistry Department, College of Science, King Saud University, Riyadh, 11451, Saudi Arabia;

^2^Department of Biomedical Sciences for Health, University of Milan, Segrate-Milano;

^3^Centre of Excellence for Nutrition (CEN), Potchefstroom, 2531, South Africa;

^4^IRCCS Fondazione Don Carlo Gnocchi, Milano;

^5^Department of Physiopathology and Transplants, University of Milano, Milano;

^6^ IRCCS Istituto Ortopedico Galeazzi, Milano.

**^§^**These authors have contributed equally to this study

*Corresponding Author:

Cecilia Gelfi, Professor

Department of Biomedical Sciences for Health, University of Milan, Via Fratelli Cervi 93, Segrate

(Milan), Italy.

Tel: +39 02 50330475.

Fax: +39 02 21717558.

e-mail: [cecilia.gelfi@unimi.it](mailto:cecilia.gelfi@unimi.it)

**A**

Optical density (AU)

nmol

**B**

Optical density (AU)

nmol

**C**

**
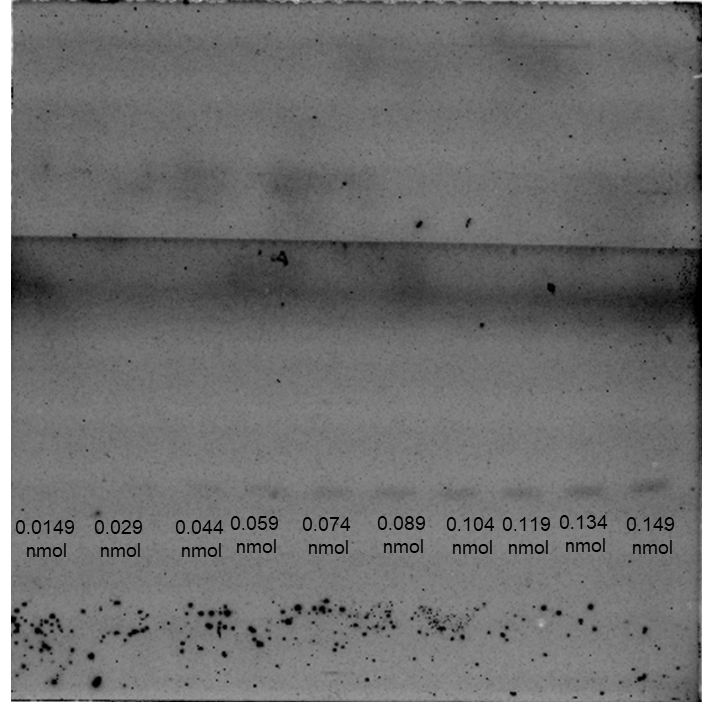
**

**D**

**
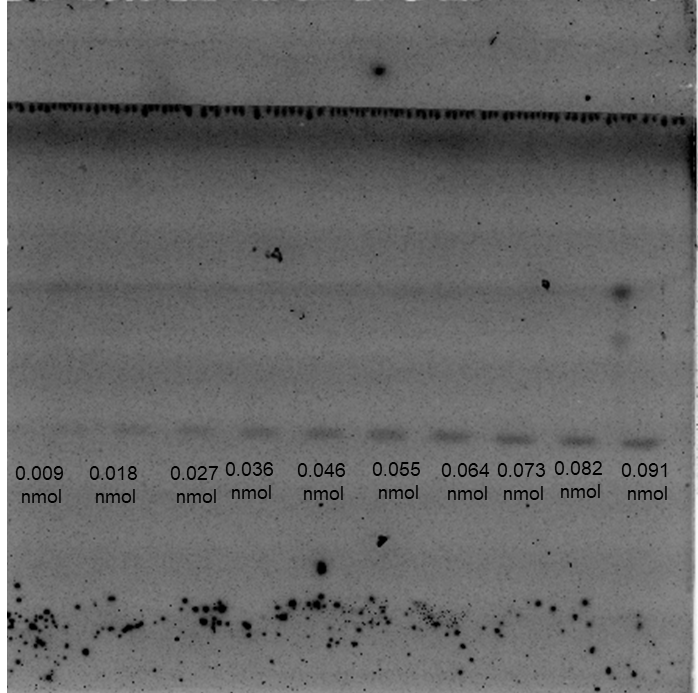
**

**Figure 1S** (A) Calibration curve obtained from 3 different HPTLC of ceramide C16:0, developed in three different days in chloroform/methanol 55:3 (v/v) and sprayed with primuline. (B) Calibration curve obtained from 3 different HPTLC of lactosylceramide C17:0, developed in three different days in chloroform/methanol/water 55:20:3 (v/v) and sprayed with primuline. (C) Limit of detection for CerC16:0 were set at 44 pmol and at 18 pmol for LacCer C17 (D).

**A**


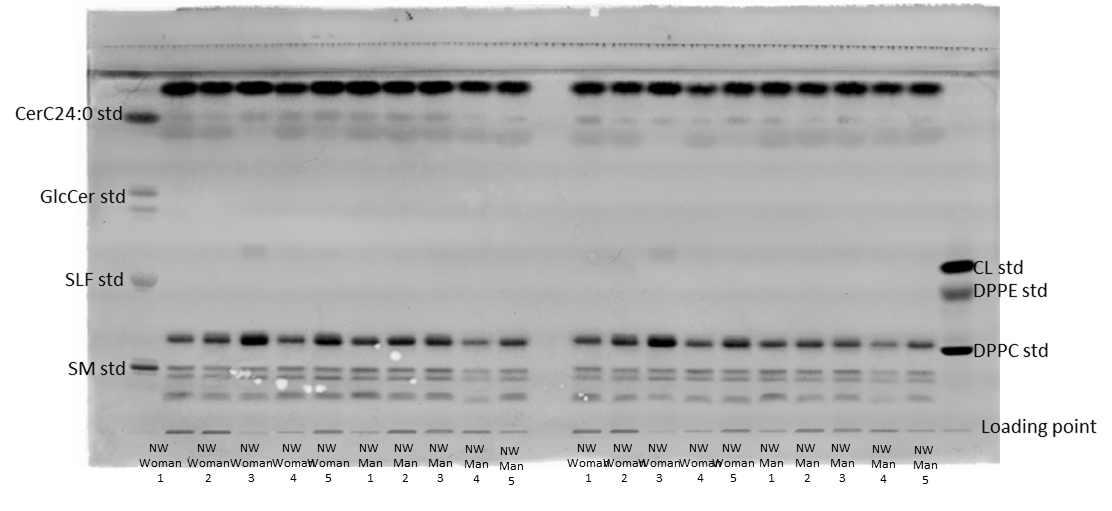


**Figure 2S**. Example of standards and samples distribution over HPTLC plate. (left) Standards from bottom to top: Sphingomyelin st (SM ST, Rf=0.176), Sulfatides (SLF St, Rf=0.436), D-glucosyl-ß-1,1'-N-stearoyl-D-erythro-sphingosine-d5 (GlcCer St, Rf=0.715) and 1,2-Dipalmitoyl-D-erythro-sphingosine (Cer C24:0 St, Rf=0.923); (right) standards from bottom to top: 1,2-Dipalmitoyl-sn-Glycero-3-Phosphocholine (DPPC St, Rf=0.218), 1,2-Dipalmitoyl-sn-Glycero-3-Phosphoethanolamine (DPPE St, Rf=0.388) and Cardiolipin (CL St, Rf=0.474) standards. Sample bands with Rf=0.923, Rf=0.858, Rf=0.176 and Rf=0.152 were respectively attributed to long chain Cers, short chain Cers, long chain SMs and short chain SMs.


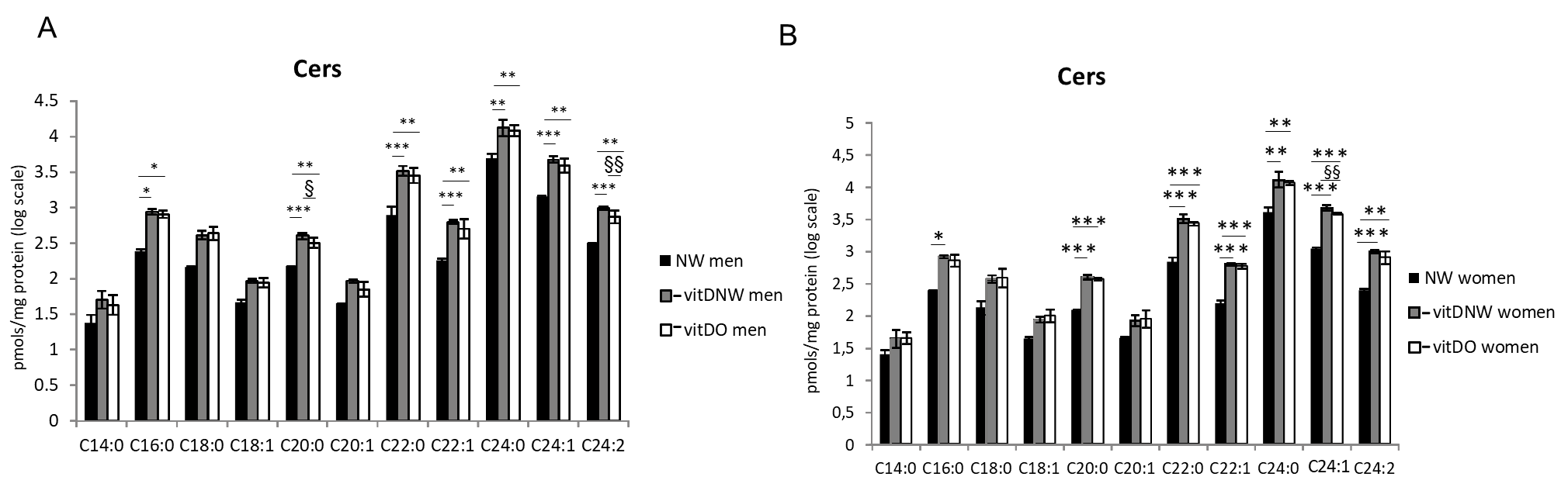

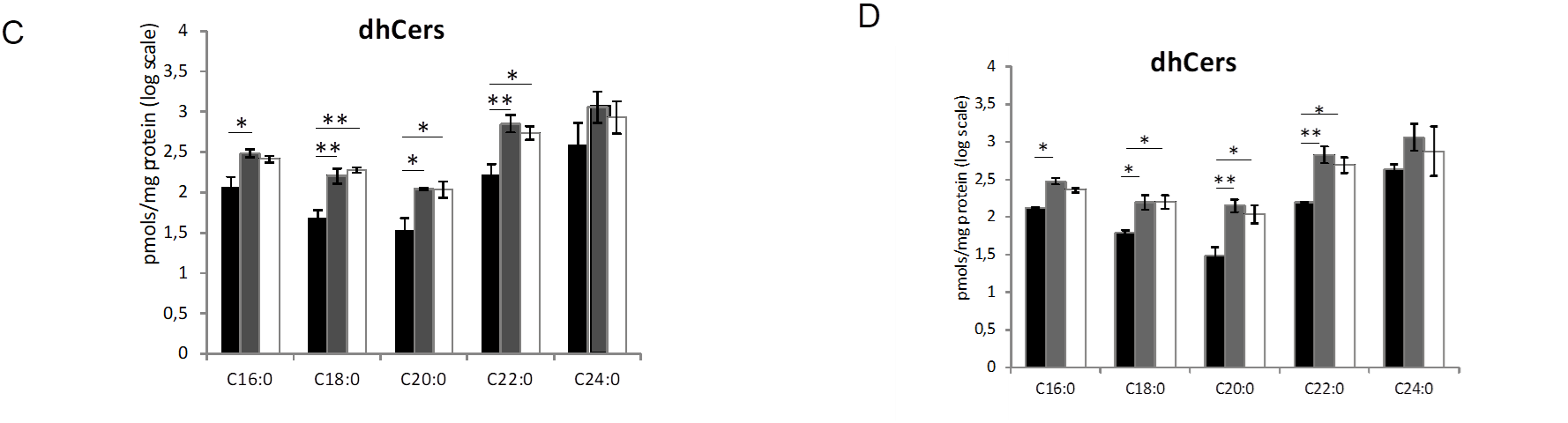

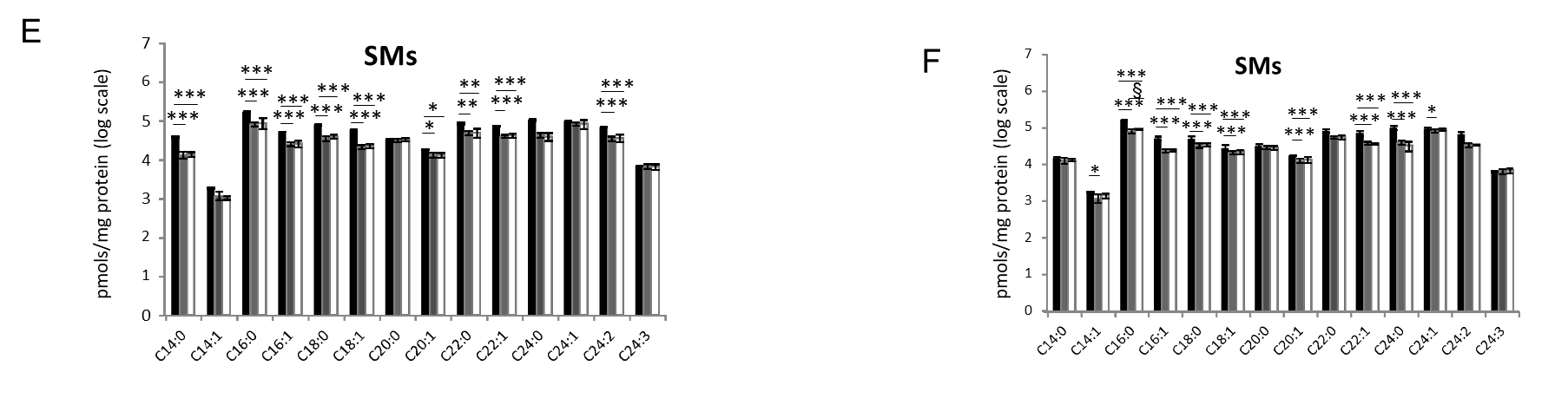

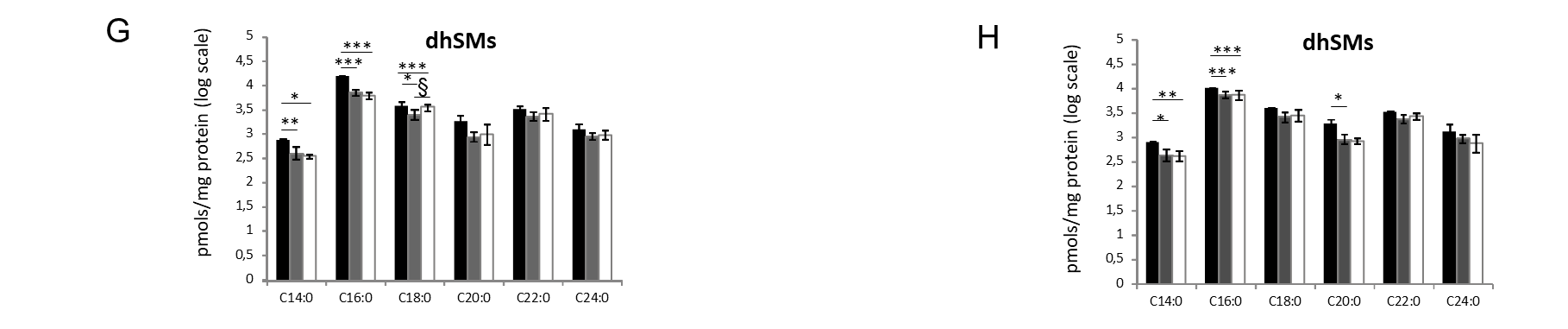


Figure 3S Cers, dhCers, SMs and dhSMs levels in sub-pooled sera from NW (black bars) vs –vitDNW (grey bars) vs -vitDO (white bars) men (A, C, E, G) and women (B, D, F, H) by LC-MS analysis. Statistical analysis was performed by ANOVA test, with Tukey post-hoc test. *symbol was used to indicate the difference vs NW, and § symbol was used to indicate the statistical difference between –vitDO and –vitDNW. Data are expressed in log scale and reported as mean ± SD.


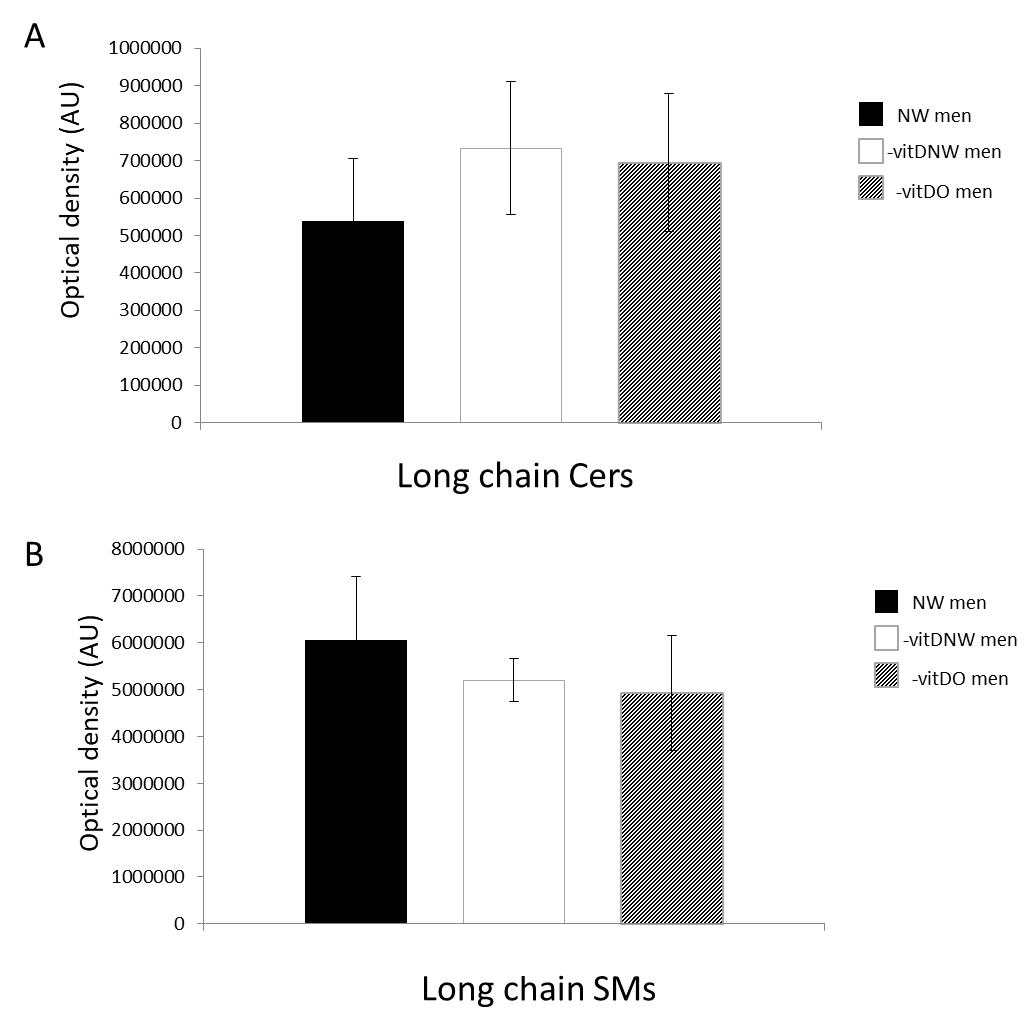


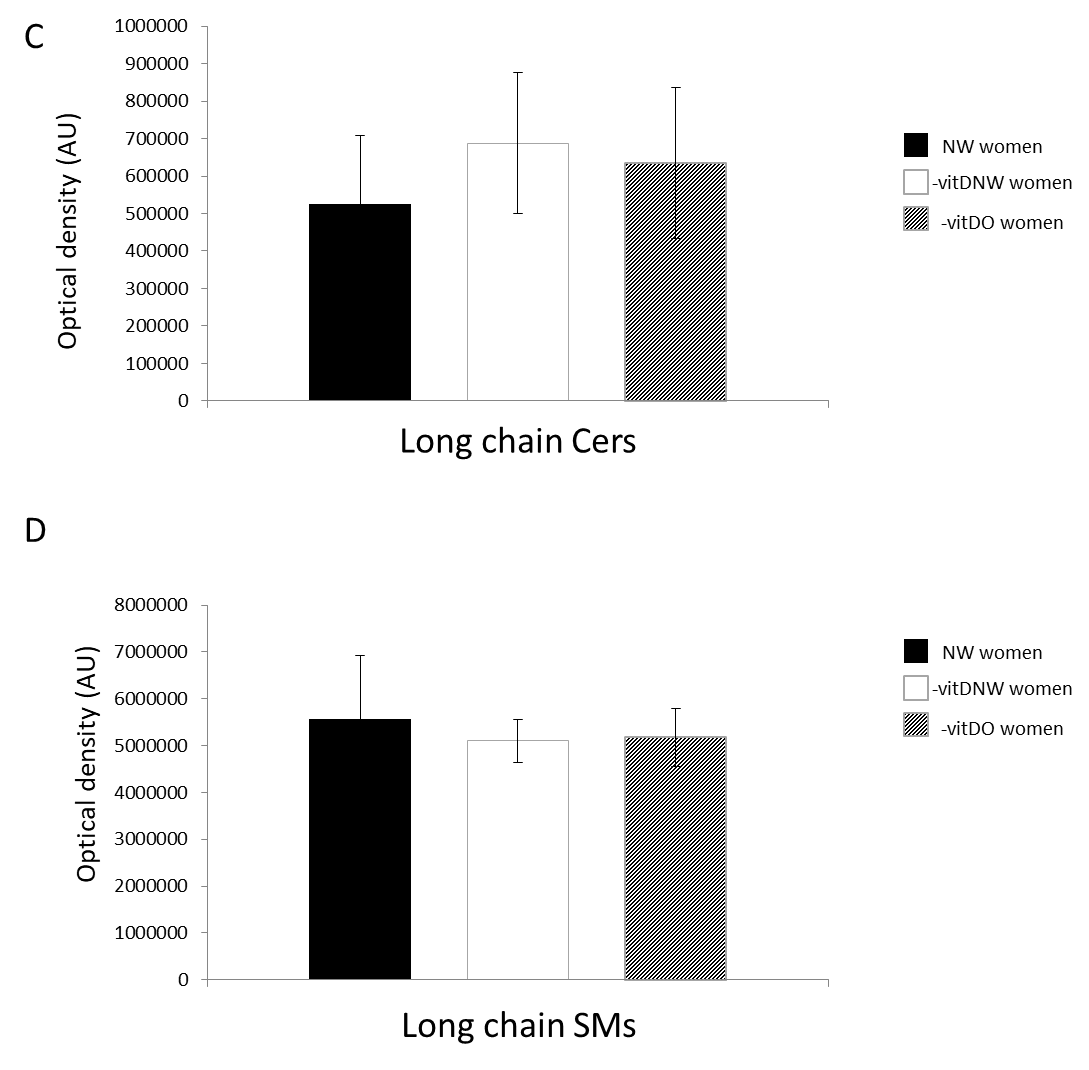


**Figure 4S** (A, B) Comparison of ceramides (Cers) C20-C24 (A) and sphingomyelins (SMs) C20-C24 (B) circulating levels in sera from normolipidemic normal weight (NW) (n=15), vitamin D deficient dyslipidemic normal weight (-vitDNW) (n=23) and vitamin D deficient obese (-vitDO) (n=25) men by primuline /HPTLC densitometry.

(C, D) Comparison of Cers C20-C24 (C) and SMs C20- C24 (D) circulating levels in sera from NW (n=8), -vitDNW (n=23) and -vitDO (n=35) women by primuline /HPTLC densitometry.

**Figure 5S** Sphingolipid biosynthetic pathway. Abbreviations: dhS1P, dihydrosphingosine-1-phosphate; SPHK, sphingosine kinase; DhCer, dihydroCeramide; dhSM, dihydrosphingomyelin; S1P, sphingosine-1-phosphate; SMase, sphingomyelinase; SM, sphingomyelin; GCase, glucosylceramidase; HexCer, hexosylceramidase; diHexCer, dihexosylceramidase.

-vitDNW men

-vitDNW women

pmols/mg protein (log scale)

-vitDO men

-vitDO women

pmols/mg protein (log scale)

pmols/mg protein (log scale)

-vitDNW men

-vitDNW women

pmols/mg protein (log scale)

-vitDO men

-vitDO women

pmols/mg protein (log scale)

pmols/mg protein (log scale)

-vitDNW men

-vitDNW women

pmols/mg protein (log scale)

pmols/mg protein (log scale)

-vitDNW men

-vitDNW women

pmols/mg protein (log scale)

-vitDO men

-vitDO women

pmols/mg protein (log scale)

pmols/mg protein (log scale)

-vitDNW men

-vitDNW women

pmols/mg protein (log scale)

-vitDNW men

-vitDNW women

pmols/mg protein (log scale)

-vitDO men

-vitDO women

pmols/mg protein (log scale)

**Figure 6S** (extension of Figure 2 and of Figure 4A, B) Comparison of SLs and GSLs circulating levels in men vs women in the three groups (NW, -vitDNW, -vitDO) by LC-MS analysis. Data are expressed in log scale.
